# Targeting DNA-PKcs and ATM with *miR-101* Sensitizes Tumors to Radiation

**DOI:** 10.1371/journal.pone.0011397

**Published:** 2010-07-01

**Authors:** Dan Yan, Wooi Loon Ng, Xiangming Zhang, Ping Wang, Zhaobin Zhang, Yin-Yuan Mo, Hui Mao, Chunhai Hao, Jeffrey J. Olson, Walter J. Curran, Ya Wang

**Affiliations:** 1 Department of Radiation Oncology, Emory University School of Medicine, Winship Cancer Institute of Emory University, Atlanta, Georgia, United States of America; 2 Department of Neurosurgery, Emory University School of Medicine, Winship Cancer Institute of Emory University, Atlanta, Georgia, United States of America; 3 Medical Microbiology, Immunology & Cell Biology, School of Medicine, Southern Illinois University, Springfield, Illinois, United States of America; 4 Department of Radiology, Emory University School of Medicine, Winship Cancer Institute of Emory University, Atlanta, Georgia, United States of America; 5 Department of Pathology and Laboratory Medicine, Emory University School of Medicine, Winship Cancer Institute of Emory University, Atlanta, Georgia, United States of America; University Medical Center Hamburg-Eppendorf, Germany

## Abstract

**Background:**

Radiotherapy kills tumor-cells by inducing DNA double strand breaks (DSBs). However, the efficient repair of tumors frequently prevents successful treatment. Therefore, identifying new practical sensitizers is an essential step towards successful radiotherapy. In this study, we tested the new hypothesis: identifying the miRNAs to target DNA DSB repair genes could be a new way for sensitizing tumors to ionizing radiation.

**Principal Findings:**

Here, we chose two genes: *DNA-PKcs* (an essential factor for non-homologous end-joining repair) and *ATM* (an important checkpoint regulator for promoting homologous recombination repair) as the targets to search their regulating miRNAs. By combining the database search and the bench work, we picked out *miR-101*. We identified that *miR-101* could efficiently target DNA-*PKcs* and *ATM* via binding to the 3′- UTR of *DNA-PKcs* or *ATM* mRNA. Up-regulating *miR-101* efficiently reduced the protein levels of DNA-PKcs and ATM in these tumor cells and most importantly, sensitized the tumor cells to radiation *in vitro* and *in vivo*.

**Conclusions:**

These data demonstrate for the first time that miRNAs could be used to target DNA repair genes and thus sensitize tumors to radiation. These results provide a new way for improving tumor radiotherapy.

## Introduction

Ionizing radiation (IR) kills cells by inducing DNA double strand breaks (DSBs), which is one of the major cancer therapy approaches. However, the efficient repair of DNA DSBs in the tumors makes the tumors radioresistant, which frequently prevents successful treatment. Therefore, identifying new practical sensitizers is an essential step towards successful radiotherapy. Mammalian cells require two major DNA DSB repair pathways: non-homologous end-joining (NHEJ) and homologous recombination repair (HRR). The human cell lines that are deficient in the DNA-PK catalytic subunit (DNA-PKcs) or ATM are sensitive to IR because of the inefficient DNA DSB repair [1], [2]. DNA-PKcs is a major factor for NHEJ [3], [4], [5], [6] and ATM, an important multi-functional protein [7], mainly promotes HRR [8], [9]. MicroRNAs (miRNAs) represent a newly discovered class of small non-coding RNAs with ∼22 nucleotides. miRNAs bind to the 3′-untranslated region (UTR) of multiple target mRNAs and either block the target translation or initiate the target degradation [10], [11], [12]. Most mammalian mRNAs are conserved targets of miRNAs [13], and it is reasoned that identifying the miRNAs that target DNA DSB repair genes could be a new way of sensitizing tumors to IR. In this study, we were interested in testing the new hypothesis that targeting *DNA-PKcs* and *ATM* with one miRNA could sensitize tumor cells to IR.

## Results and Discussion

### Chose *miR-101* that could bind to the 3′-UTR of *DNA-PKcs* and *ATM*

**Figure 1:**
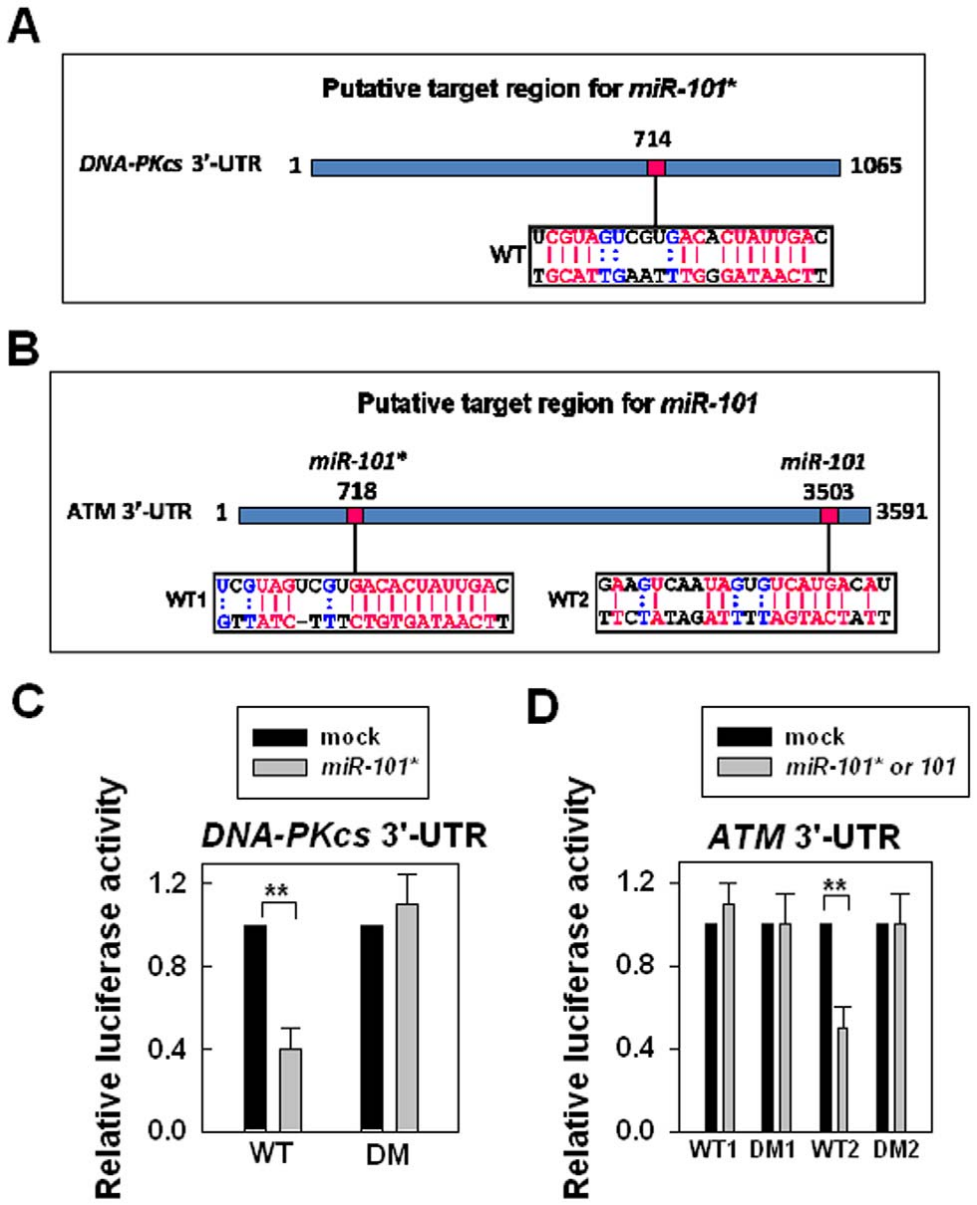
The effects of the putative *miR-101* binding sites in the 3′-UTR of *DNA-PKcs* or *ATM* on the luciferase activity. (A) The putative miR-101* binding site in the 3′-UTR of *DNA-PKcs* (WT). (B) The putative miR-101* or *miR-101* binding sites in the 3′-UTR of *ATM* (WT1, WT2). (C) The effects of miR-101*-binding site in the 3′-UTR of *DNA-PKcs* on the luciferase activity. 293T cells were transfected with the firefly luciferase reporter plasmid containing partial 3′-UTR of *DNA-PKcs* with the putative miR-101* binding site (WT) or without the binding site (DM). Luciferase activity was assayed 48 h after transfection with the *miR-101* mimic (*miR-101*) or without the mimic (mock), **, *p*<0.01. (D) The effects of miR-101* or *miR-101*-binding sites in the 3′-UTR of *ATM* on the luciferase activity. 293T cells were transfected with the firefly luciferase reporter plasmid containing partial 3′-UTR of *ATM* with the putative miR-101* (WT1) or *miR-101* (WT2) binding site or without the binding site (DM1, DM2). Luciferase activity was assayed 48 h after transfection with the *miR-101* mimic (*miR-101*) or without the mimic (mock), **, *p*<0.01.

To identify a miRNA that could efficiently target both *DNA-PKcs* and *ATM*, we combined the database (miRBase, microcosm Targets Version 5 and miRanda) search and the manual check for the matched sequences. As a result, we found that *miR-101* is one of the candidates because the duplex (miR-101* and *miR-101*) contains the matched sequences to the 3′-UTR of *DNA-PKcs* or *ATM* mRNA (Figure 1A, B). To examine whether DNA-PKcs or ATM is a target of *miR-101*, we cloned the partial 3′-UTR of *DNA-PKcs* or *ATM* mRNA (∼300 bp) containing a wild type or deleted mutant *miR-101* or miR-101*-binding sequence (Figure 1A, B) downstream of the firefly luciferase reporter gene. We examined the effects of *miR-101* on the luciferase activity at these regions by using a mimic *miR-101* RNA that contains duplex strands of *miR-101* (including *miR-101* and miR-101*). The results showed that the luciferase activity was significantly suppressed by the reporter containing the wild type 3′-UTR of *DNA-PKcs* (WT) but was not affected by the reporter without the binding site: DM (Figure 1C). In addition, the luciferase activity was significantly suppressed by the reporter containing the wild type 3′-UTR of *ATM* (WT2) but was not affected by the reporter containing the other wild type 3′-UTR of *ATM* (WT1) or the reporter without the binding site (DM1 or DM2) (Figure 1D). These data suggest that *miR-101* could suppress the expression of DNA-PKcs or ATM through the binding sequence at the 3′-UTR of *DNA-PKcs* (WT) by the strand, miR-101* or at the 3′-UTR of *ATM* (WT2) by the strand, *miR-101*.

### Identify DNA-PKs and ATM as the targets of *miR-101*

**Figure 2:**
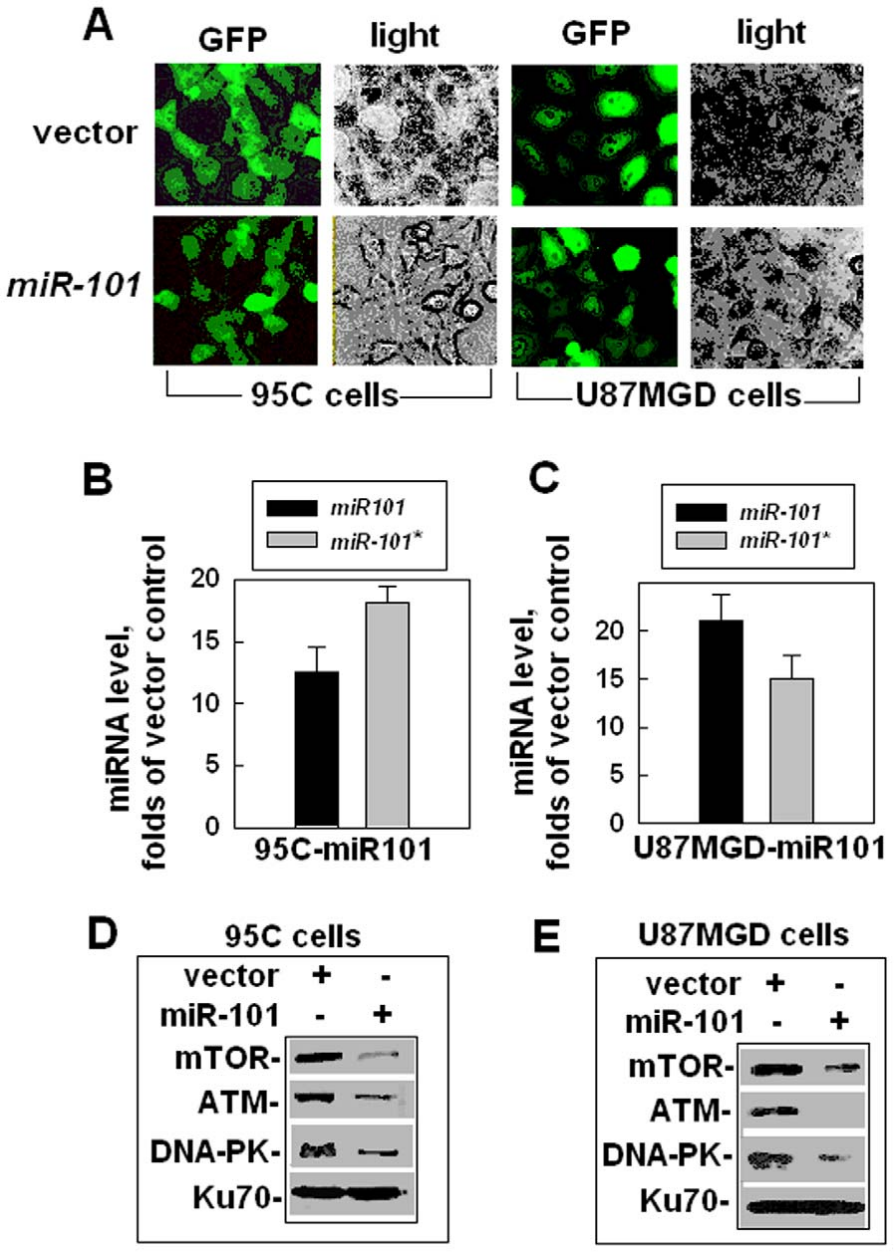
DNA-PKs and ATM as the targets of *miR-101*. (A) Up-regulating *miR-101* in 95C cells and U87GMD cells. The images reflect GFP signals, which represent the infection efficiencies of the lentivirus vectors. (B) The *miR-101* level (including both strands: *miR-101* and miR-101*) was measured by qRT-PCR in 95 C cells. (C) The *miR-101* level (including both strands: *miR-101* and miR-101*) was measured by qRT-PCR in U87GMD cells. (D) The effects of up-regulation of *miR-101* on DNA-PKcs and ATM expression in 95C cells. Ku70 was used as an internal loading control. (E) The effects of up-regulation of *miR-101* on DNA-PKcs and ATM expression in U87GMD cells. Ku70 was used as an internal loading control.

To verify whether DNA-PKcs or ATM is a target of *miR-101*, we made a lentiviral construct that contained a precursor of *miR-101* (Figure S1). We used the lentiviral construct containing a *pri*-*miR-101* to transfect different cell lines in two different ways: 1. We transfected one pair of human lung cancer cell lines: 95C and 95D cells with the *miR-101* vector and a vector encoding the antibiotic marker, we selected the antibiotic resistant colonies from the transfected cells; 2. We used the lentiviral construct with the viral helper to infect one human GBM cell line, U87MGD cells, and collected the cells at 48–72 h after infection. (The data derived from the 95D cells over-expressed with *miR-101* were similar to that from the 95C cells up-expressed with *miR-101*, therefore, we showed one set of results from 95C cells in this manuscript only.) The exogenous *miR-101* expressed well in both 95C-miR101 and U87MGD-miR101 cells (Figure 2A). qRT-PCR confirmed that the exogenous *miR-101* including both strands: *miR-101* and miR-101*, over-expressed in 95C (Figure 2B) and U87MGD cells (Figure 2C). The result from the RNase protection experiments provided additional evidence that *miR-101* over-expressed in the cells transfected with the lentiviral vector encoding *miR-101* (Figure S2). The levels of the three PI-3 kinase like kinase (PIKK) family members: DNA-PKcs, ATM (we predicted in this study) and mTOR (reported by another group [14]) were dramatically decreased in both cell lines: 95C-miR101 cells and U87MGD-miR101 cells, when compared with that in their counterparts (Figure 2D, E). These results indicate for the first time that, besides mTOR, DNA-PK and ATM are also targets of *miR-101*. The results concerning the auto-phosphorylation level of DNA-PKcs or ATM (Figure S3) provided additional evidence to support this conclusion.

### Up-regulating *miR-101* sensitizes tumor cells to radiation

**Figure 3:**
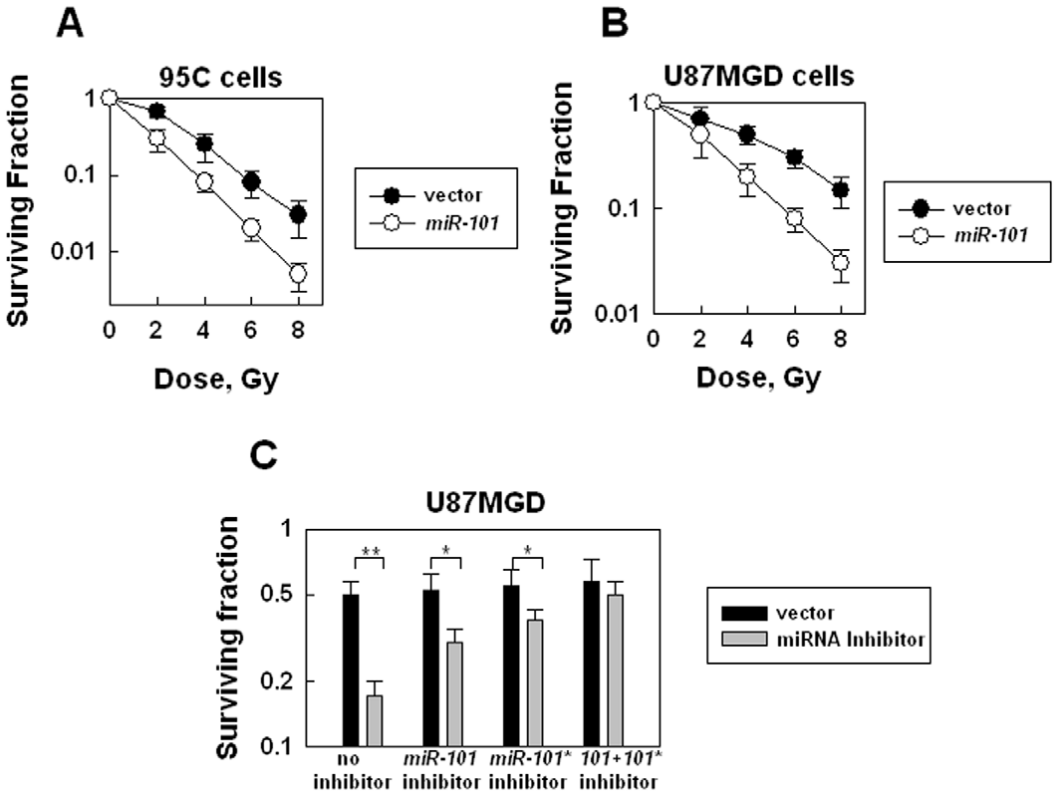
Effects of up-regulation of *miR-101* on the cell radiosensitivity. (A) The effect of up-regulation of *miR-101* on 95C cell radiosensitivity. The clonogenic assay was performed as described in Materials and Methods. Data shown are the mean and SE from three independent experiments. (B) The effect of up-regulation of *miR-101* on U87GMD cell radiosensitivity. At 72 h after infection with the lentivirus encoding *pri-miR-101*, the cells were exposed to different doses. The clonogenic assay was performed as described above. Data shown are the mean and SE from three independent experiments. (C) The effects of the *miR-101* or miR-101* inhibitor on the sensitivity of the *miR-101* over-expressed U87MGD cells to IR. At 48 h after infection with the lentivirus encoding *pri-miR-101*, the cells were transfected with the inhibitor for an additional 36 h. The cells were exposed to 4 Gy and the clonogenic assay was performed as described above. Data shown are the mean and SE from three independent experiments, **, *p*<0.01.

To examine the effects of *miR-101* on the sensitivities of theses tumor cell lines to IR, we performed the clonogenic assay. The results showed that the cells over-expressed with *miR-101* were much more sensitive to IR than their counterparts (Figure 3A, B). The *miR-101*-induced sensitization levels in these cell lines are 2.5–5 folds at different dose points. The inhibitor of *miR-101* (targeting ATM but not DNAPKcs) or miR-101* (targeting DNA-PKcs but not ATM) partially reversed the sensitivity of the cells over-expressed with *miR-101*, and combining the two inhibitors almost completely reversed the cell sensitivity (Figure 3C), confirming that the sensitization effects are derived from both strands of *miR-101*, which target both DNA-PKcs and ATM. *miR-101* targets the three members in the PIKK family: DNA-PKcs, ATM and mTOR. To determine whether mTOR, similar to DNA-PK and ATM, also contributed to the sensitization of the cells to IR, we examined the sensitivity of the cells to IR after the mTOR level was knocked down by a siRNA or the mTOR activity was inhibited by rapamycin in the cells. The results showed that when mTOR was down-regulated by siRNA (Figure S4A) or the mTOR activity was inhibited by rapamycin in the cells (Figure S4B), the sensitivity of the cells to IR did not change (Figure S4C, D). These results confirm that over-expressing *miR-101*-induced cell radio-sensitization is independent of mTOR. Our recent data about another miRNA, *miR-100* that could also sensitize the cells to IR by targeting ATM (our unpublished data) provided additional evidence to support that targeting DNA DSB repair genes could sensitize the cells to IR-induced killing.

### Up-regulation of *miR-101* sensitizes human xenografts to radiation

**Figure 4:**
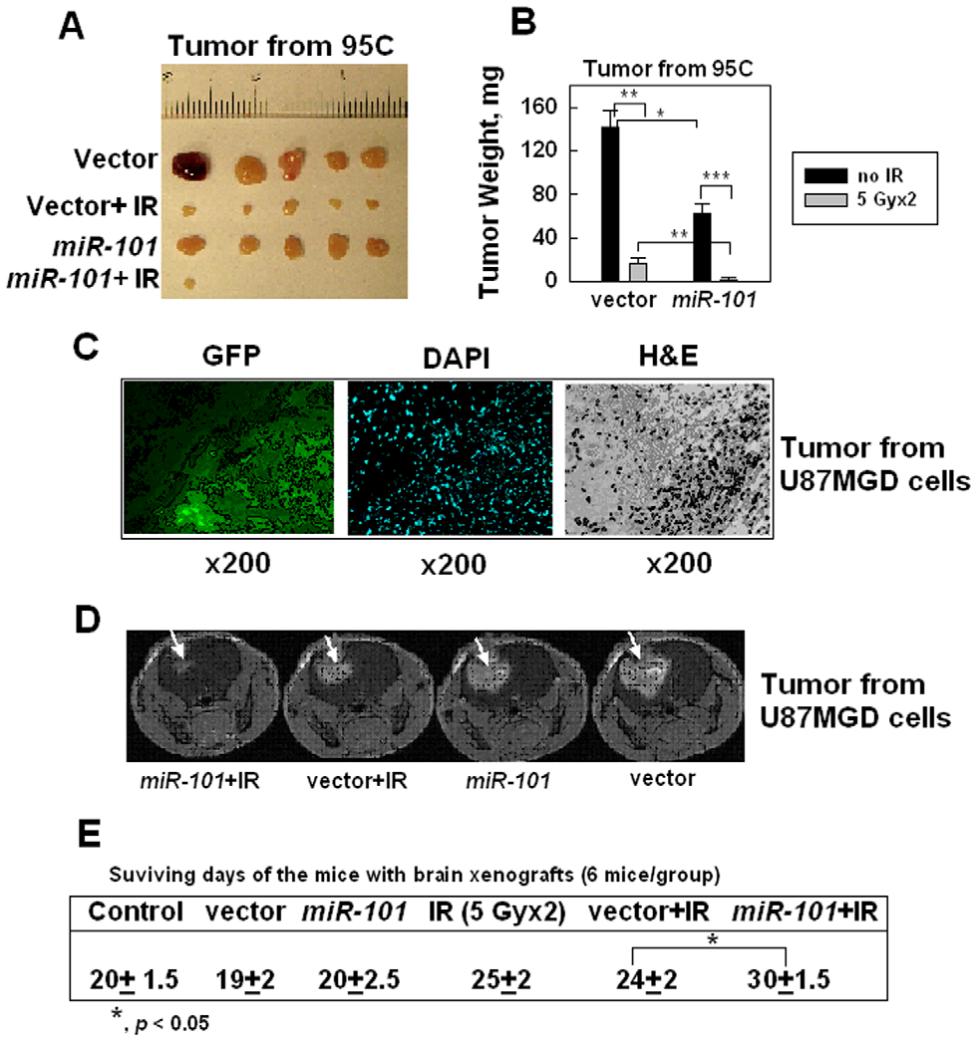
Effects of up-regulation of *miR-101* on the xenograft radiosensitivity. (A) Tumor size reflected the effects of *miR-101* on the subcutaneous tumor radiosensitivity. Both hind legs of each *nu/nu* mouse were injected with the 95C cells with or without *miR-101* up-regulated (5 mice were injected with the *miR-101* up-regulated 95C cells and 5 mice were injected with the vector-transfected 95C cells, 10 mice total). The right hind leg that born the developed tumor was exposed to IR (5 Gy×2, at 72 h interval) at 12 days after the tumor cell injection and the left hind leg that born the developed tumor was used as the mock-irradiated control. The mice were sacrificed at 21 days after the tumor cell injection and the tumors were removed for weight comparison. (B) Tumor weight reflected the effects of *miR-101* on the subcutaneous tumor radiosensitivity. The data shown are the mean and SE: *, *p*<0.05; **, *p*<0.01; ***, *p*<0.001. (C) Brain tumor that developed from U87MGD glioma cells and injected with the lentiviral vector. At 72 h after the viral vector was injected, the mice were sacrificed and the brain tissues were prepared for the pathological slides. The GFP signals were detected by a fluorescence microscope from the frozen samples. H&E staining was used for distinguishing the tumor and normal brain tissue from the formalin-fixed samples. (D) MRI reflected the effects of *miR-101* on the brain tumor radiosensitivity. MRI scans of individual mouse brain 18 d after intracranial inoculation of U87GMD cells. The presence of a glioma (white arrows) was detected as the bright areas with an MRI contrast agent (Gd-DTPA). (E) Survival days reflected the effects of *miR-101* on the brain tumor radiosensitivity. The data shown are the mean and SE; *, *p*<0.05.

To study whether *miR-101* could sensitize tumors to IR, we first compared the growth rates between 95C-miR101 and 95C-vector cells because it was recently reported that over-expression of *miR-101* could inhibit hepatocellular carcinoma development [15]. The results showed that 95C-miR101 cells did grow slowly the first 2 days after plating when compared with 95C-vector cells, however, the two cell lines did not show apparent differences in their growth rates after 2 days, both in an exponential style (Figure S5). These results allowed us to use the cells developing xenograft in mice and to examine the sensitivities of the tumors to IR *in vivo*. Next, we injected 95C-miR101 or 95C-vector cells to both hind legs of each mouse: 5 mice and 10 tumors for each type cells (10 mice in total). The tumors in the mice derived from the cells were observed at ∼10 days after the cell inoculation. We then irradiated one hind leg of each mouse including the tumor area (5 Gy, 2 times at 72 h intervals) at 12 days after the tumor cell inoculation and the other hind leg including the tumor area was used as the mock-irradiation control. The results showed that without irradiation, the size of the xenografts in mice hind legs derived from 95C-miR101 cells was smaller than that from 95C-vector cells at 21 days when we ended the experiment (Figure 4A,B), indicating that *miR-101* inhibited tumor growth, which is consistent with other report [15]. More importantly, the results showed that the size of the xenograft derived from 95C-miR101 cells after receiving the radiation (5 Gy×2) was much smaller than that from 95C-vector cells after receiving the same doses of radiation (Figure 4A, B). These results indicate that *miR-101* could sensitize tumors to radiation when *miR-101* is pre-over-expressed in tumors.

To study whether up-regulation of *miR-101* by delivering lentiviral vector encoding *miR-101* to the tumor site (post tumor-forming) could sensitize the tumor to radiation, we chose the mouse brain xenografts derived from U87MGD cells because brain tumor treatment depends more on the radiotherapy than other organ tumors due to the brain-blood barrier. To avoid any possibility that *miR-101* would induce a harmful effect on the mice born with the brain tumor, we did a literature search because one miRNA could target many targets with different effects on the tumors derived from different tissues [16], [17], [18], [19]. By combining our data (Figure 4A, B) with already published information that *miR-101* targets many oncogenes such as mTOR [14], E2H2 [20], Mcl-1 [15], [21], and FOS [15], [21], and is low-expressed in many tumors, we believe that over-expressing *miR-101* will not promote tumor growth. We then injected the tumor cells into the mice brains. At 10 days after the tumors formed in the mice brain, we delivered the lentiviral vector containing *miR-101* to the brain tumor site. To confirm that the lentiviral vector was delivered to the tumor site, we sacrificed 2 mice at 72 h after the vector delivery and removed the brain tissue for frozen or formalin-fixed sample preparation. The results showed that the GFP signal was limited to the tumor area (Figure 4C), indicating that the lentiviral vector was delivered to the tumor site and did not spread to normal tissues. We irradiated (5 Gy) the mouse head including the tumor area at 72 h after delivering the lentiviral vector. The mouse head was irradiated (5 Gy) again at 72 h. We examined the brain tumor size with magnetic resonance imaging (MRI) at 3 days after IR (5 Gy×2). The results showed that there was no apparent difference in the tumor sizes delivered with lentiviral vector with or without *miR-101* (Figure 4D), however, the tumor size with lentiviral vector encoding *miR-101* plus IR (5 Gy×2) was smaller than that with lentiviral vector alone plus IR (Figure 4D). The survival results showed that the mice dying at ∼20 days after the tumor cells were injected in their brains without IR (Figure 4E) and the mice with the lentiviral vector or the vector encoding *miR-101* showed a similar survival time (Figure 4E). These data provide additional evidence that the lentiviral vector did not stimulate the tumor growth. IR (5 Gy×2, at 72 h interval) extended the mice' survival days to ∼25 days (Figure 4E), indicating that IR delayed the tumor growth. Although delivering the lentiviral vector alone at 72 h before IR did not extend the mice' survival days (Figure 4E), delivering the lentiviral vector containing *miR-101* at 72 h before IR clearly extended the mice' survival days to ∼30 days (Figure 4E). The body weight of these mice at 2 days after IR provided additional evidence that demonstrated the radio-sensitizing effects of *miR-101* on the tumors (Figure S6). These results strongly support that *miR-101* could be delivered to the tumor site and sensitize the tumor to radiation.

The field of small RNAs is rapidly developing toward *in vivo* delivery for therapeutic purposes. Although it was reported that miRNAs could serve as potential agents and alter resistance to cytotoxic anticancer therapy [22]; until now, there is no report that uses one miRNA to directly target the DNA repair gene and sensitize tumors to radiation or chemotherapy. Our data in this study demonstrate for the first time the feasibility. The biggest advantage for choosing miRNAs as a therapeutic tool is that one miRNA could target multi-targets with less degradation due to the Drosha-Dicer modification process in the cell. Advanced molecular therapy aimed at up-modulating the level of a given miRNA in the mouse model has been reported with a different viral vector [19]. We believe that our data demonstrate that CHOSING THE INTERESTING GENES AND THEN IDENTIFING THE MATCHED MIRNAS TO TARGET THE GENES provides a new strategy for future miRNA-therapy.

## Materials and Methods

### Ethics Statement

All the mouse work was followed using the approved animal protocol according to the guidelines of Emory University Institutional Animal Care and Use Committee. The protocol number is 004-2010 and the title of the protocol is “Study the effects of miRNA on sensitizing malignant glioma cells to radiation therapy”. All mice were handled in strict accordance with good animal practice as defined by the relevant national and/or local animal welfare bodies.

### Plasmids construction

To construct a plasmid expressing *miR-101*, we amplified *pri-miR-101* using the genomic DNA from a healthy blood donor as we previously did for the *miR-145* construction [23] only with the different primers (Supplementary Table S1). The amplified fragment was first cloned into a PCR cloning vector and subsequently cloned into a lentiviral vector: pCDHCMV-MCS-EF1-copGFP (System Biosciences) at the *Eco*R1 and *Not*I sites. Expression of *miR-101* was verified by TaqMan real-time RT-PCR. The luciferase-UTR reporter plasmid that contains the *DNA-PKcs* or *ATM* 3′-UTR carrying a putative *miR-101* or miR-101* binding site (WT for DNA-PKcs, WT1 or WT2 for ATM) or a deleted mutant without the *miR-101* or miR-101* binding site (DM for DNA-PKcs, DM1 or DM2 for ATM) was constructed as follows: Briefly, the complementary oligonucleotides (Table S1) for each selected regions were hybridized to form double-stranded DNA and inserted into pMIR-ReporterTM firefly luciferase vector (Applied Biosystems, Foster City, CA, USA). All constructs were confirmed by sequencing.

### PCR/RT-PCR and quantitative RT-PCR (qRT-PCR)

PCRs were performed to amplify pri-microRNA sequences or the *DNA-PKcs* or *ATM* 3′-UTR sequence according to the standard three-step procedure. For RT-PCR, total RNA was isolated by using a Trizol reagent (Invitrogen) and the small RNA was isolated by using a miRNeasy Mini Kit (Qiagen). RNA (1 µg) was used to synthesize cDNA by using a TaqMan® MicroRNA Reverse Transcription Kit (Applied Biosystems). qRT-PCR was performed in triplicate with a TaqMan® Universal PCR Master Mix and a specific TaqMan® MicroRNA assay (Applied Biosystems) on an ABI PRISM® 7000 Sequence Detection System (Applied Biosystems). Samples were normalized to RNU48 RNA, and relatively quantified using a 2^−ΔΔC^_T_ method [24].

### RNase protection assay

The RNA probes were constructed by PCR and *in vitro* transcription. Briefly, forward and reverse primers that include a T7 promoter upstream to a mature miRNA sequence (*hsa-miR-101* or RNU48) with 10 over-lapping nucleotides were designed (Supplementary information Table S1). Amplified PCR was purified by using a QIAquick spin column (Qiagen, Valencia, CA, USA) and proceeded with a Megashortscript™ kit (Ambion, Austin, TX, USA) according to the manufacturer's protocol. The RNA probes were hybridized to the total RNA of U87MGD cells infected with the lentiviral vector or the vector encoding *miR-101* by using a mirVana™ miRNA detection kit (Ambion) according to the manufacturer's instructions. Gel was exposed directly to a phosphor screen overnight and the signals were detected by using a Typhoon™ 9210 (GE, Bio-Sciences, Piscataway, NJ, USA).

### Cell lines and transfection/transduction

The lung cancer cell lines, 95C and 95D were obtained from Dr. Yinglin Lu's laboratory at the 301 Hospital, Beijing China [25]. 293FT cells were purchased from the American Type Culture Collection. The human GMB cell line, U87MGD, was obtained from Dr. Van Meir's laboratory at Emory University, Atlanta, USA [26]. 95C or 95D cells were directly co-transfected with the lentiviral vector-miR101 and the pCDHCMV-MCS-EF1 plasmid encoding a puromycin (Puro) antibiotic selective marker (System Biosciences), at a ratio of 20∶1 by using Lipofectamine 2000 (Invitrogen) according to the manufacturer's instructions. The Puro resistant colonies were selected and the *miR-101* levels were measured by qRT-PCR. The glioma cell lines: U87MG or M059K cells were transduced by the packaged lentivirus. Briefly, approximately 2×10^6^ 293FT cells were seeded in a 100 mm dish overnight. The lentiviral vector-*miR-101* or lentiviral vector alone (2 µg) and pPACKH1 Packaging Plasmid Mix (10 µg) (System Biosciences, Mountain View, CA, USA) were formed into a complex with Lipofectamine™ 2000 and transfected to the 293FT cells. The culture medium containing the packaged viruses was harvested at 48 hr after transfection and spun at 4°C, 3000 rpm for 10 min. The supernatant was collected and polybrene was added to the final concentration 8 µg/ml. The mixture (5 ml) was added to the glioma cell culture in a 100 mm dish with 5 ml of medium. The transduced cells were harvested after 72–96 hr post-infection for further experiments. For siRNA and miRNA inhibitor transfection, Lipofectamine™ 2000 was mixed with either 100 nM siRNA of *ATM*, *Dicer* (Santa Cruz Biotech Inc), *hsa-miR-101* inhibitor, or hsa-miR-101* inhibitor (Thermo Fisher Scientist Inc) as previously described [27]. Cells were harvested at 36 hr after transfection for further experiments.

### Antibodies and reagents

The anitibody against DNA-PKcs (MS-370-P1) was purchased from Thermo Fisher Scientific Inc. The antibodies against ATM (2837S), mTOR (2927), p70 S6 kinase (9202) and phospho-p70 S6 kinase at Thr389 (9202) were purchased from Cell Signaling. The antibody against autophosphorylated DNA-PKcs (S2056) was kindly provided by Dr. Benjamin P. C. Chen at the UT Southwestern Medical Center [28]. The antibody against autophosphorylated ATM (S1981) was purchased from Rockland Inc. The antibody against Ku70 (SC-17789) was purchased from Santa Cruz Biotech Inc.

### Luciferase assay

293FT cells were transfected with the appropriate plasmids with or without 100 nM *hsa-miR-101* mimics (Thermo Fisher Scientific) in 48-well plates. The cells were harvested 48 h after transfection, the cells were then lysed with a luciferase assay kit (Promega) according to the manufacturer's protocol and were measured on a luminescence microplate reader LUMIstar Galaxy (BMG labtechnologies). β-galactosidase or renilla luciferase was used for normalization.

### Cell radiosensitivity assay

Cell sensitivity to radiation was determined by the loss of colony-forming ability. Briefly, the cells were irradiated by using an x-ray machine (X-RAD 320, N. Branford, CT, USA) at 320 kV, 10 mA, with a filtration of 2-mm aluminum. The dose rate was 2 Gy/min. After IR, the cells were collected and plated, aiming at a density of 20–100 colonies per dish. Two replicate dishes were prepared for each datum point, and cells were incubated for 2 weeks. Colonies were stained with crystal violet (100% methanol solution).

### Xenograft tumor radiosensitivity studies

For subcutaneous xenografts, briefly, both hind legs of each nu/nu mouse were subcutaneously injected with 2×10^6^ 95C cells transfected with vector alone or with the vector encoding *miR-101*, 5 mice for each group. Ten days later, when the xenografts formed in both hind legs, the right hind leg of each mouse was exposed to x-ray (5 Gy) and the left one was used as the mock-irradiation control. The right hind legs were irradiated with 5 Gy again at 72 h. The radiation was performed by the same x-ray machine with a different filter (1.5 mm aluminum, 0.8 mm tin and 0.25 mm copper), at a dose rate of 1 Gy/min. The mice were sacrificed at 21 days after the tumor cell inoculation and the tumors were removed and weighted. For brain xenografts, briefly, 2.5×10^5^ glioma cells were stereotactically injected into the brains of athymic nu/nu mice. A 2-mm drill was then used to make a burr hole 2 mm to the right and 1 mm anterior of the bregma of the skull. A 23-gauge Hamilton syringe was advanced 2.5 mm deep, and then retracted 0.5 mm for implanting 5 µL of tumor cell suspension. Ten days later, the mouse brain tumor site was injected with 5 µL of lentiviral vector (pCDH-CMV-MCS-EF1-copGFP) with or without *miR-101*. At 72 h after the lentiviral vector injection, the mouse head including the brain tumor area was exposed to x-ray (5 Gy), and irradiation were repeated with 5 Gy at 72 h. The body weight was determined at 18 days after the tumor cell inoculation.

### Magnetic resonance imaging (MRI)

Magnetic resonance imaging (MRI) was performed on a 4.7T MRI scanner (Philips Intera) using a small volume coil (4 cm diameter). Matrix of 256 (reconstructed to 512) and 0.5 or 1 mm slice thickness were used to collect a set of axial images (typically 15–20 slices). T2 weighted fast spin echo imaging using parameters of TR/TE = 5000/56ms and T1 weighted spin echo imaging using TR/TE = 400/11ms were applied. The average number of signals was typically set at 4 to obtain sufficient signal to noise ratio. An MRI contrast agent, gadolinium diethylenetriamine-pentaacetic acid (Gd-DTPA), was administrated (i.v.) at a dose of 0.2 mmol/kg and followed by a post-contrast T1 weighted spin echo imaging by using the parameter above.

### Statistical analysis

Statistical analysis of data was done using the Student's *t* test. Differences with *p*<0.05 are considered significant.

## Supporting Information

Table S1 Primer information.
(0.05 MB DOC)

Figure S1 The plasmid map. The pri-miR-101 using genomic DNA from a healthy blood donor as a template was amplified. The PCR reactions were performed with the specific primers (Table S1) by using a high fidelity Phusion enzyme (New England Biolabs). The amplified fragment was first cloned into a PCR cloning vector and subsequently cloned into pCDHCMV-MCS-EF1-copGFP at the EcoRI and NotI sites.
(1.27 MB TIF)

Figure S2 Different expression of miR-101. Different expression of miR-101 in U87MGD cells with or without vector infection was detected by using an RNase protection assay. 1, 2: U87MGD cells without infection; 3, 4: U87MGD cells infected with the lentiviral vector encoding miR-101; 5, 6: U87MGD cells infected with the lentiviral vector alone. Lanes 1, 3, 5: the RNAs were amplified by PCR with the RNU48 primers, and the RNU48 RNA was used as the internal loading controls; 2, 4, 6: the RNAs were amplified by PCR with the miR-101 primers.
(0.69 MB TIF)

Figure S3 Effects of miR-101 on the autophosphorylation of ATM or DNA-PKcs. 95C cells transfected with the vector alone or encoding miR-101 were exposed to ionizing radiation (4 Gy), and were returned to the 37°C incubator. At 1 h after radiation, the cells were collected for preparing whole cell lyses. The autophosphorylational signals of ATM S1981 (p-ATM) or DNA-PKcs S2056 (p-DNA-PKcs) were detected by Western blot. Ku70 was used as an internal loading control.
(0.80 MB TIF)

Figure S4 Effects of mTOR on cell radiosensitivity. (A) 95C cells were transfected with mTOR siRNA (100 nM) or control RNA. The cells were collected at 48 h after transfection and the protein levels were detected by Western blot. Ku70 was used as an internal loading control. (B) The cells were treated with rapamycin 20 nM for 30′ in a serum free condition and were added with equal medium containing 20% calf serum for 3 h. The cells were collected and the protein levels were detected by Western blot. Ku70 was used as an internal loading control. (C) The cells were irradiated at 48 h after transfection with the RNA and the clonogenic assay was performed. The data represent mean and SE of three independent experiments. (D) The cells were treated with rapamycin as described in (B) and were irradiated with different doses. The clonogenic assay was performed. The data represent mean and SE of three independent experiments.
(1.50 MB TIF)

Figure S5 Effects of miR-101 on the cell growth. 95C cells transfected with the vector alone or encoding miR-101 were plated into 60 mm dishes with 20,000 cells, 5 dishes for each group. The cells were collected and counted with a Coulter Counter. The data represent mean and SE of three independent experiments.
(1.07 MB TIF)

Figure S6 Effects of miR-101 on the body weight of the mice born with the brain tumor derived from U87MGD cells. The head of the mice were injected with U87MGD cells. The mice were divided into 6 groups (6 mice/group): 1. no-treatment; 2. the brain tumor site was injected with lentiviral vector alone 10 days after tumor cell implantation, 3. the brain tumor site was injected with lentiviral vector encoding miR-101; 4. the head of the mice born with the tumor was irradiated (5 Gy×2, at 72 h interval) at 10 days after the tumor cell inoculation; 5. at 72 h after the vector without miR-101 injection, the head of the mice born with the tumor was irradiated (5 Gy×2); 6. at 72 h after the vector with miR-101 injection, the head of the mice born with the tumor was irradiated (5 Gy×2). The mice were weighed at 18 days after the tumor cell inoculation. The data represent mean and SE of six mice for each group: *, p<0.05 and **, p<0.01.
(1.17 MB TIF)
