# Supplementary material for: Targeting DNA-PKcs and ATM with miR-101 Sensitizes Tumors to Radiation
Source: PLoS One. 2010 Jul 1;5(7):e11397. doi: 10.1371/journal.pone.0011397 (PMC2895662; doi:10.1371/journal.pone.0011397)
Supplement: Table S1 — Primer information. (0.05 MB DOC) [file pone.0011397.s001.doc]

| **Primer name** | **Sequence 5'-3'** | **Remark** |
| --- | --- | --- |
| Pri-miR-101 Forward: | AATCATGCAGTTGTTCATCC |  |
| Pri-miR-101 reverse: | CCCATGTTACAAAACAAGGC |  |
| DPK101Ast-P714WTU | CTAGCTTTGCATTGAATTTGGGATAACTTCAA | Wild-type |
| DPK101Ast-P714WTL | AGCTTTGAAGTTATCCCAAATTCAATGCAAAG |  |
| DPK101Ast-P714DeU | CTAGACATAAAAGTGCTTCAAAAATCCCATGG | Mutant |
| DPK101Ast-P714DeL | AGCTCCATGGGATTTTTGAAGCACTTTTATGT |  |
| ATM101-P3504WTU | CTAGATTTTCTATAGATTTTAGTACTATTGAA | Wild-type |
| ATM101-P3504WTL | AGCTTTCAATAGTACTAAAATCTATAGAAAAT |  |
| ATM101-P3504DeU | CTAGTGAAATTATCTATTGAATGTATTACTTT | Mutant |
| ATM101-P3504DeL | AGCTAAAGTAATACATTCAATAGATAATTTCA |  |
| ATM101Ast-P718WTU | CTAGTATGTTATCTTTCTGTGATAACTTCAT | Wild-type |
| ATM101Ast-P718WTL | AGCTATGAAGTTATCACAGAAAGATAACATA |  |
| ATM101Ast-P718DeU | CTAGTACTGTCCATGTATCATAGATTGCCTTC | Mutant |
| ATM101Ast-P718DeL | AGCTGAAGGCAATCTATGATACATGGACAGTA |  |
| Hub-actin-F | TGACCCAGATCATGTTTGAGACCT | Housekeeping gene |
| Hub-actin-R | GACTCCATGCCCAGGAAGGAAG |  |
| ATM-rtF | CAGCTTGATGAGGATCGAACAGAG | RT-PCR |
| ATM-rtR | TGTGCACCATTCAAGAACACCACT |  |
| T7miR101F | CGGTTAATACGACTCACTATAGGGAGATTCAG |  |
| miR101R | TACAGTACTGTGATAACTGAATCTCC |  |
| T7RNUF | CGGTTAATACGACTCACTATAGGGAGAGGTCAGA |  |
| RNU48F | GGTCAGAGCGCTGCGGTGAT |  |
| RNU48R | GATGACCCCAGGTAACTCTGAGTGTGT |  |
