# Supplementary figures and images for: Targeting DNA-PKcs and ATM with miR-101 Sensitizes Tumors to Radiation
Source: PLoS One. 2010 Jul 1;5(7):e11397. doi: 10.1371/journal.pone.0011397 (PMC2895662; doi:10.1371/journal.pone.0011397)

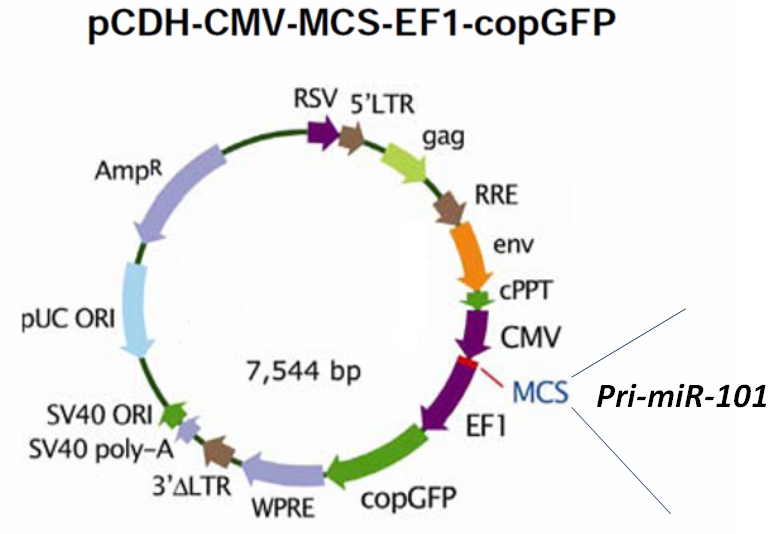

Supplement: Figure S1 — The plasmid map. The pri-miR-101 using genomic DNA from a healthy blood donor as a template was amplified. The PCR reactions were performed with the specific primers (Table S1) by using a high fidelity Phusion enzyme (New England Biolabs). The amplified fragment was first cloned into a PCR cloning vector and subsequently cloned into pCDHCMV-MCS-EF1-copGFP at the EcoRI and NotI sites. (1.27 MB TIF) [file pone.0011397.s002.tif]

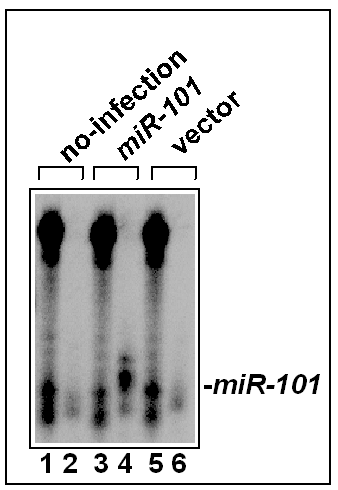

Supplement: Figure S2 — Different expression of miR-101. Different expression of miR-101 in U87MGD cells with or without vector infection was detected by using an RNase protection assay. 1, 2: U87MGD cells without infection; 3, 4: U87MGD cells infected with the lentiviral vector encoding miR-101; 5, 6: U87MGD cells infected with the lentiviral vector alone. Lanes 1, 3, 5: the RNAs were amplified by PCR with the RNU48 primers, and the RNU48 RNA was used as the internal loading controls; 2, 4, 6: the RNAs were amplified by PCR with the miR-101 primers. (0.69 MB TIF) [file pone.0011397.s003.tif]

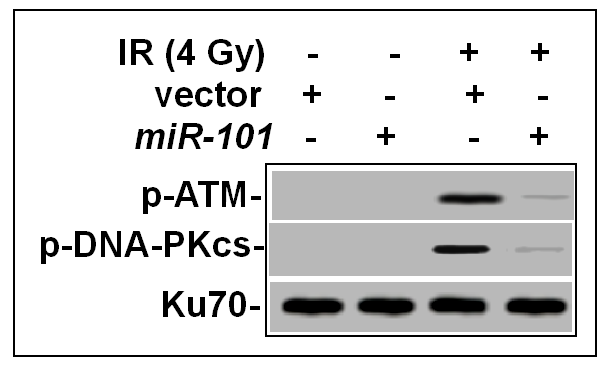

Supplement: Figure S3 — Effects of miR-101 on the autophosphorylation of ATM or DNA-PKcs. 95C cells transfected with the vector alone or encoding miR-101 were exposed to ionizing radiation (4 Gy), and were returned to the 37°C incubator. At 1 h after radiation, the cells were collected for preparing whole cell lyses. The autophosphorylational signals of ATM S1981 (p-ATM) or DNA-PKcs S2056 (p-DNA-PKcs) were detected by Western blot. Ku70 was used as an internal loading control. (0.80 MB TIF) [file pone.0011397.s004.tif]

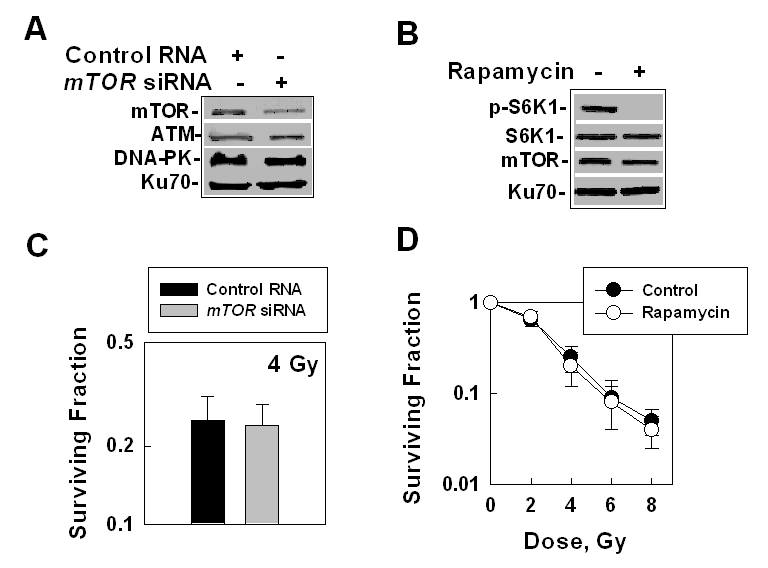

Supplement: Figure S4 — Effects of mTOR on cell radiosensitivity. (A) 95C cells were transfected with mTOR siRNA (100 nM) or control RNA. The cells were collected at 48 h after transfection and the protein levels were detected by Western blot. Ku70 was used as an internal loading control. (B) The cells were treated with rapamycin 20 nM for 30′ in a serum free condition and were added with equal medium containing 20% calf serum for 3 h. The cells were collected and the protein levels were detected by Western blot. Ku70 was used as an internal loading control. (C) The cells were irradiated at 48 h after transfection with the RNA and the clonogenic assay was performed. The data represent mean and SE of three independent experiments. (D) The cells were treated with rapamycin as described in (B) and were irradiated with different doses. The clonogenic assay was performed. The data represent mean and SE of three independent experiments. (1.50 MB TIF) [file pone.0011397.s005.tif]

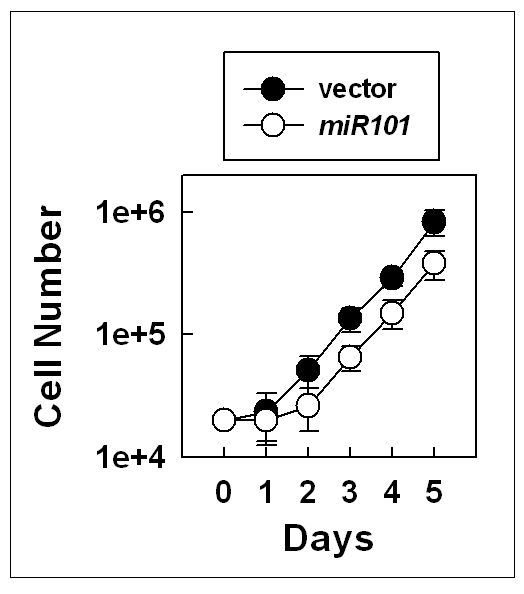

Supplement: Figure S5 — Effects of miR-101 on the cell growth. 95C cells transfected with the vector alone or encoding miR-101 were plated into 60 mm dishes with 20,000 cells, 5 dishes for each group. The cells were collected and counted with a Coulter Counter. The data represent mean and SE of three independent experiments. (1.07 MB TIF) [file pone.0011397.s006.tif]

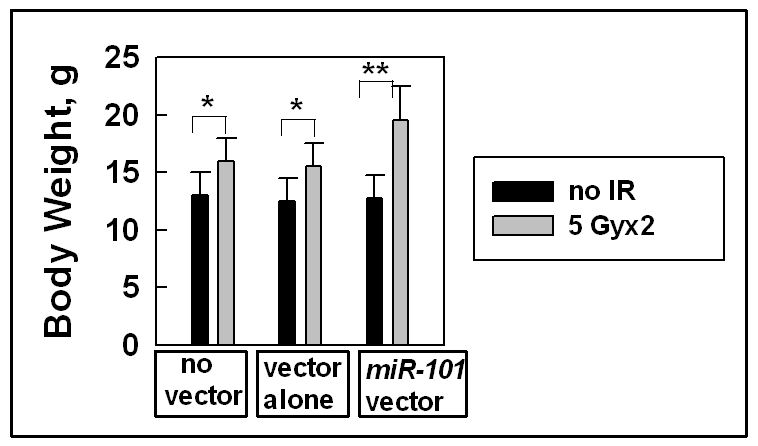

Supplement: Figure S6 — Effects of miR-101 on the body weight of the mice born with the brain tumor derived from U87MGD cells. The head of the mice were injected with U87MGD cells. The mice were divided into 6 groups (6 mice/group): 1. no-treatment; 2. the brain tumor site was injected with lentiviral vector alone 10 days after tumor cell implantation, 3. the brain tumor site was injected with lentiviral vector encoding miR-101; 4. the head of the mice born with the tumor was irradiated (5 Gy×2, at 72 h interval) at 10 days after the tumor cell inoculation; 5. at 72 h after the vector without miR-101 injection, the head of the mice born with the tumor was irradiated (5 Gy×2); 6. at 72 h after the vector with miR-101 injection, the head of the mice born with the tumor was irradiated (5 Gy×2). The mice were weighed at 18 days after the tumor cell inoculation. The data represent mean and SE of six mice for each group: *, p<0.05 and **, p<0.01. (1.17 MB TIF) [file pone.0011397.s007.tif]
